# Insight into the cold adaptation and hemicellulose utilization of *Cladosporium neopsychrotolerans* from genome analysis and biochemical characterization

**DOI:** 10.1038/s41598-018-24443-7

**Published:** 2018-04-17

**Authors:** Rui Ma, Huoqing Huang, Yingguo Bai, Huiying Luo, Yunliu Fan, Bin Yao

**Affiliations:** 1 Key Laboratory for Feed Biotechnology of the Ministry of Agriculture, Feed Research Institute, Chinese Academy of Agricultural Sciences, Beijing, China; 2 Biotechnology Institute, Chinese Academy of Agricultural Sciences, Beijing, China

## Abstract

The occurrence of *Cladosporium* in cold ecosystems has been evidenced long before, and most of the knowledge about nutrient utilization of this genus is sporadic. An alpine soil isolate *C. neopsychrotolerans* SL-16, showing great cold tolerance and significant lignocellulose-degrading capability, was sequenced to form a 35.9 Mb genome that contains 13,456 predicted genes. Functional annotation on predicted genes revealed a wide array of proteins involved in the transport and metabolism of carbohydrate, protein and lipid. Large numbers of transmembrane proteins (967) and CAZymes (571) were identified, and those related to hemicellulose degradation was the most abundant. To undermine the hemicellulose (xyaln as the main component) utilization mechanism of SL-16, the mRNA levels of 23 xylanolytic enzymes were quantified, and representatives of three glycoside hydrolase families were functionally characterized. The enzymes showed similar neutral, cold active and thermolabile properties and synergistic action on xylan degradation (the synergy degree up to 15.32). Kinetic analysis and sequence and structure comparison with mesophilic and thermophilic homologues indicated that these cold-active enzymes employed different cold adaptation strategies to function well in cold environment. These similar and complementary advantages in cold adaptation and catalysis might explain the high efficiency of lignocellulose conversion observed in SL-16 under low temperatures.

## Introduction

The large fraction of the Earth’s surface, up to 80%, is occupied by permanently cold ecosystems (below 5 °C), which include deep sea as well as polar (Arctic and Antarctic) and alpine regions^1^. Although such habitats experience extreme conditions that are challenging to most life forms, various microorganisms that colonize these cold environments have adapted to low temperatures. Based on their temperature tolerance, Morita^2^ has grouped these microorganisms into psychrophilic or psychrotolerant: psychrophiles can grow at or below 0 °C and have optimum growth temperatures of ≤15 °C and maximum cardinal temperatures of ≤20 °C, while psychrotolerants have optimum and maximum growth temperatures of >15 °C and >20 °C. However, when extending to higher organisms, such as algae, plants, insects, fish and invertebrates, these definitions are ambiguous and inappropriate, thus Cavicchioli^3^ introduced ecological terms stenopsychrophile (psychrophile) and eurypsychrophile (psychrotolerant) to differ organisms with narrow and wide growth temperature ranges, respectively.

To survive in extremely cold environments, microorganisms display some common molecular and physiological characteristics, including increased fluidity of cellular membranes, ability to accumulate compatible solutes, expression of cold shock, antifreeze and ice-nucleating proteins, production of cold-active enzymes, and rapid horizontal gene transfer exerted by plasmids^4–6^. And the strategies adopted by cold-adapted enzymes have been proposed, including but not limited to increased flexibility at the active site^7,8^, fewer interactions like ion pairs, aromatic interactions, hydrogen bonds and salt bridges^9^, larger and more accessible catalytic cavity^10^, alternations of amino acid composition and increased solvent-accessible area^11^, and oligomerization^12^. Unfortunately, how these cold-adapted enzymes acted altogether on biomass degradation and conversion to release fermentable sugars in cold habitats is rarely studied.

*Cladosporium* as the eurypsychrophilic fungus has been discovered in various cold environments, i.e. the oligotrophic soil^13–15^, air^16^, benthic mats^17^, marine sponges^18,19^ and sea water^20^ of the Antarctic, the alpine soil of Tibetan plateau^21^ and Changbai^22^ and Tianshan^23^ Mountains, the deep sea of Pacific Ocean^24^ and South China^25^, and bat^26^. Currently 205 species are accepted in *Cladosporium*, and only a few temperature studies were undertaken in eurypsychrophilic *Cladosporium* species^23,27–30^. *Cladosporium* is normally associated with a necrotrophic or hemibiotrophic lifestyle^31^ and has great capability of degrading lignocellulose^22,32–36^ and polycyclic aromatic hydrocarbons like phenanthrene and anthracene^37,38^. However, so for only a few Carbohydrate Active enZymes (CAZymes), including glucoamylase^39^, xylanase^40,41^ and α-galactosidase^42^, have been functionally characterized. This sporadic knowledge provides some clues to but still far away from the underlying mechanisms of lignocellulose utilization of *Cladosporium*, not to speak of eurypsychrophilic *Cladosporium*.

In our previous study, a eurypsychrophilic strain *C. neopsychrotolerans* SL-16 was isolated from the alpine soil of Tianshan Mountain^23^. To undermine its mechanism in cold adaptation and nutrient utilization, the SL-16 genome was sequenced, and the genes related to plant cell wall degradation were predicted. The genes related to hemicellulose degradation under low temperatures were diverse and abundant, and a xylanolytic enzyme system was selected for qPCR and functional analysis. To our best knowledge, it is the first report on comprehensive genomic characterization of eurypsychrophilic *Cladosporium* species. We suggest that the synergistic actions on xylan degradation and complementary advantages in cold adaptation of xylanolytic enzymes are important traits of *C. neopsychrotolerans* for hemicellulose utilization under low temperature conditions.

## Results

### Lignocellulose-degrading capability of strain SL-16

**Figure 1:**
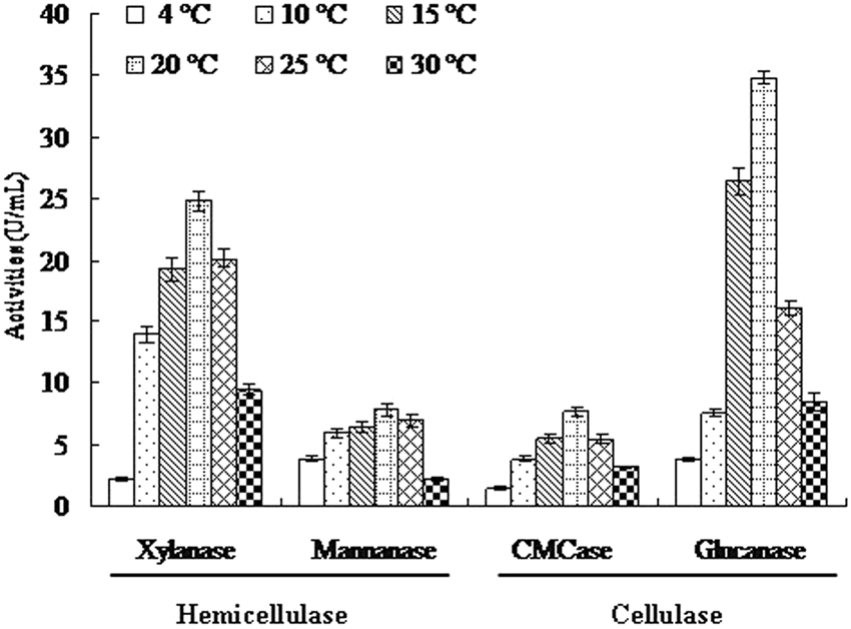
Lignocellulose-degrading activities of *C. neopsychrotolerans* SL-16 after 7-day-growth at different temperatures.

*C. neopsychrotolerans* SL-16 was initially isolated at 4 °C, and showed optimal growth at 15 °C^23^. To induce the production of lignocellulose-degrading enzymes, aliquots of spore suspension (1 × 10^7^ CFU) were inoculated into a wheat bran medium^43^. After 7-day-growth at 4–30 °C, the cultures showed significant cellulase (CMCase and glucanase) and hemicellulase (xylanase and mannanase) activities (1.47–34.8 U/mL, Fig. 1), with maxima occurred at 20 °C. It suggested that strain SL-16 is a eurypsychrophilic fungus with significant lignocellulose-degrading capability.

### Genomic features and annotation

A 500-bp insert library (4.8 Gb) was generated in the present study. After assembly the sequence data, the draft genomic DNA sequence of strain SL-16 was 35,929,705 bp in length with the GC content of 52.64% (Table 1). The sequenced reads of 4,827 Mb represents ~138-fold depth of genome sequence coverage. This project has been deposited at DDBJ/EMBL/GenBank under the accession PEGC00000000.

**Table 1:** Genome features of *C. neopsychrotolerans* SL-16^a^.

	C. neopsychrotolerans SL-16
Size of total reads (Mb)	4,827
Assembly size (bp)	35,929,705
Number of contigs (≥200 bp)	1,739
Contig size (N50) (kp)	610,333
Number of scaffolds (≥200 bp)	653
Scaffold size (N50) (bp)	936,277
G + C content (%)	52.64
Number of predicted genes	13,456
Average gene length (bp)	1,409
Average number of exons per gene	2.3
tRNA	204
KEGG	2,059
GO	7,269
KOG	4,256
NR	11,368
Swiss-Prot	5,756
TrEMBL	11,379

^a^Gene annotation was performed against the KEGG, GO, KOG, NR, Swiss-Prot and TrEMBL with the e-value of ≤1e^−5^.

**Figure 2:**
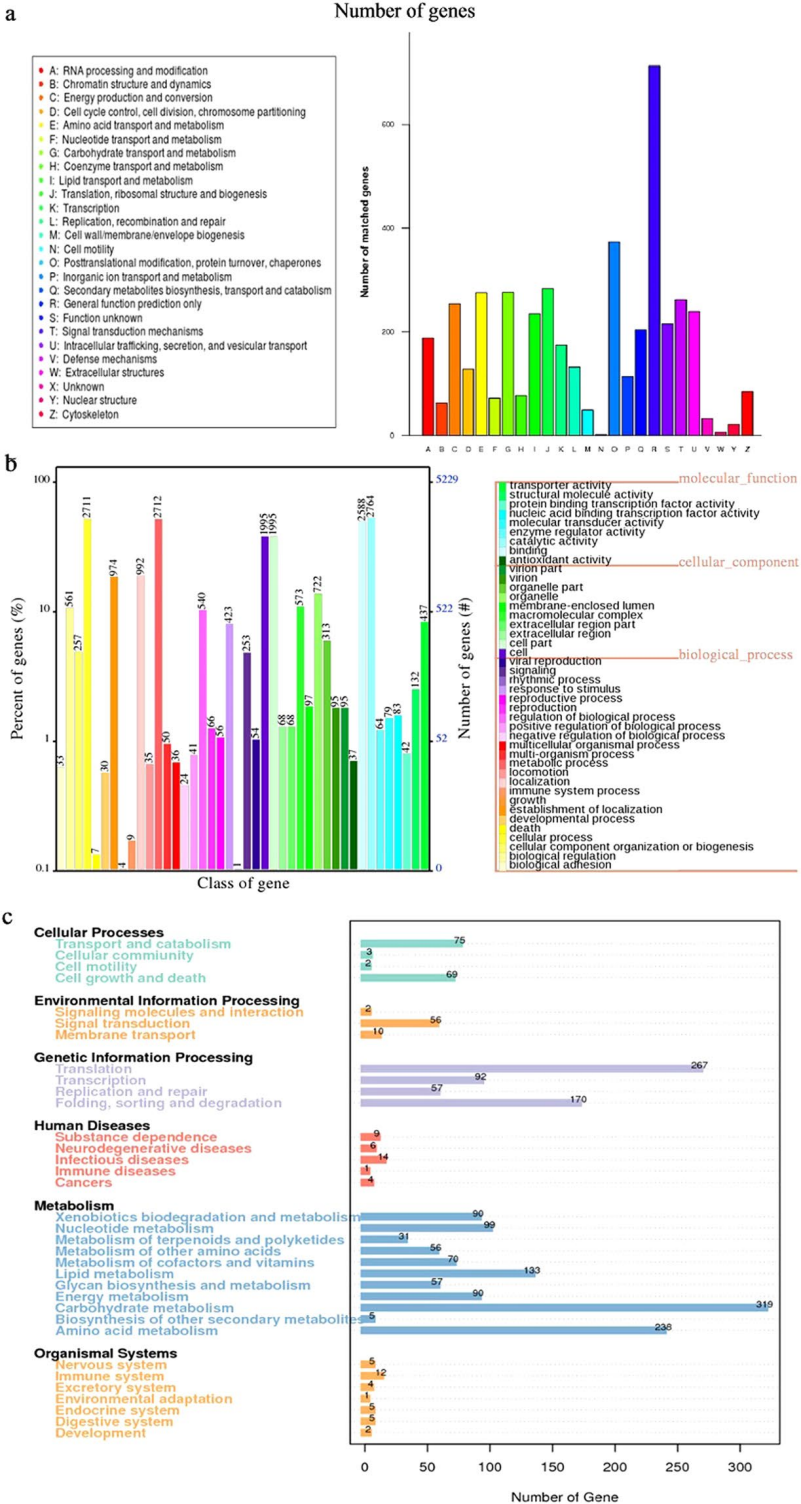
KOG (**a**), GO (**b**) and KEGG (**c**) classification of predicted genes in *C. neopsychrotolerans* SL-16.

*De novo* sequencing and homology searching predicted 13,456 genes, and 11,684 of them were mapped to the KOG, GO, KEGG, NR, Swiss-Prot and TrEMBL (Table 1; Fig. 2). A total of 4,256 predicted proteins were annotated into 25 KOG major classifications (Fig. 2a). Among the highest annotated groups, posttranslational modification, protein turnover and chaperon appeared to play significant roles in cellular regulation, development and adaptation, while transport and metabolism of amino acid and carbohydrate appeared important in nutrient absorption and utilization. Based on the GO classifications, 7,269 predicted genes were associated with 42 subcategories (Fig. 2b). Those related to cellular process (GO: 0009987) and metabolic process (GO: 0008152 and 0044238), organic substance metabolic process (GO: 0071704), catalytic activity (GO: 0003824) and binding (GO: 0005488) were highly abundant and diverse. A total of 2,059 genes were annotated into KEGG pathways (Fig. 2c), and the top three metabolic pathways were carbohydrate metabolism (319), translation (267), and amino acid metabolism (236). Further transmembrane protein prediction using the TMHMM software indicated that strain SL-16 contained 967 genes which coded for putative proteins with transmembrane α-helix structures. The presence of large numbers of protein-encoding genes related to carbohydrate and amino acid metabolism and nutrient transport may contribute to the survival and growth of SL-16 in cold environment.

### CAZymes

CAZymes are capable of degrading the plant cell wall into carbon sources for fungal growth. In this study, a total of 571 putative CAZymes were identified in strain SL-16, including 298 glycoside hydrolases (GH), 83 glycoside transferases (GT), 73 auxiliary activities (AA), 64 carbohydrate-binding modules (CBM), 34 carbohydrate esterases and 19 polysaccharide lyases (PL) (Supplementary Table S1). The CAZymes of eight biomass-degrading fungal representatives and two *Cladosporium* strains were compared with those of SL-16 (Table 2). SL-16 had advantages in the quantity of GHs (298 vs. 174–268), and these GHs showed 39–91% identities to known sequences. According to the definition of CAZymes involved in the degradation of plant cell wall^44^, SL-16 contains 235 (vs. 77–190) CAZymes involved in the degradation of hemicellulose (92), pectin (71), the side chains of hemicellulose and pectin (55), cellulose (8), and 9 carbohydrate binding domain (CBM) (Supplementary Table S2). In comparison to other biomass-degrading fungi, *Cladosporium* spp. contain a larger number of CAZymes involved in the degradation of hemicellulose and pectin, 129–218 vs. 49–124 (86.1–92.8% vs. 52.0–82.1%), confirming the preference of *Cladosporium* for soft plant tissues as a carbon source^36^. The CAZymes of SL-16 were also compared with those of the two *Cladosporium* strains (Supplementary Table S1, Table 2). SL-16 was distinguished by the high numbers of CBMs (64 vs. 28 and 41, especially the exclusively fungal CBM1 [24 vs. 0 and 12]), GHs (298 vs. 268 and 261, such as the GH3, GH5, GH11, GH43 and GH76), and PLs (19 vs. 9 and 14, especially the PL1 and PL3). In comparison to the plant pathogen *C. fulvum* and human allergen/pathogen *C. sphaerospermum*, SL-16 from barren alpine soil with more diverse CAZyme-encoding genes might have greater lignocellulose-degrading capability. Moreover, with the absence of CAZymes of CE11, GH73, GH80 and GH82, SL-16 was suggested to be associated with a necrotrophic or hemibiotrophic lifestyle^31,45^.

**Table 2:** Numbers of CAZymes of *Cladosporium* spp. and eight biomass-degrading fungi.

Strains	Accession number	Total protein	CAZymes	AAs	CBMs	CEs	GHs	GTs	PLs
*C. neopsychrotolerans* SL-16	PEGC00000000	13,456	571	73	64	34	**298**	83	19
*C. fulvum* CBS13901	AMRR00000000	**14,127**	445	—	28	35	268	**105**	9
*C. shaerospermum* UM843	AIIA00000000.2	9,562	**605**	77	41	**114**	261	98	14
*Aspergillus fumigatus* TIGR	GCA_000002655.1	9,630	486	38	64	26	257	86	15
*Aspergillus nidulans* ASM1142v1	GCA_000011425.1	10,527	504	50	56	29	266	81	**22**
*Chaetomium globosum* CBS 148.51	GCA_000143365.1	11,048	541	**100**	**96**	38	220	72	15
*Myceliophthora thermophila* ATCC 42464	GCA_000226095.1	8,548	393	57	53	25	184	66	8
*Neurospora crassa*	GCA_000182925.2	9,701	375	52	58	23	174	64	4
*Penicillium chrysogenum* ASM71027v1	GCA_000710275.1	11,090	450	55	53	18	225	90	9
*Trichoderma reesei*	GCA_000167675.2	9,849	359	32	39	15	195	74	4
*Thielavia terrestris* NRRL 8126	GCA_000226115.1	9,801	450	68	65	25	213	75	4

#### Xylanolytic gene expression upon xylan induction

Strain SL-16 was distinct due to the high number of putative hemicellulases (92 vs. 31–78; Supplementary Table S2). Xylan is the main component of hemicellulose, thus its degrading enzymes were selected to undermine the nutrient utilization of SL-16 under cold conditions. Upon the induction by wheat bran medium^43^ at 15 °C, a xylan main chain-degrading system (Table 3) consisting of 5 GH10 xylanases, 8 GH11 xylanases, and 10 xylosidases-encoding genes was selected for mRNA quantification. Such a multifunctional xylanolytic enzyme system is found to be quite common among fungi^46–48^, in which different enzyme components cooperate in xylan degradation. The cDNA fragments coding for the mature enzymes and reference β-tubulin were amplified with specific primers and used as templates for the generation of standard curves. Standard curves of *C*_*t*_ vs. cDNA concentration for each gene were generated (Supplementary Table S3), with the PCR efficiencies ranging from 96.7% to 106.5%. The relative fold change of each gene was determined after normalization to that of *β-tubulin* (Table 3). Analysis of the transcription patterns indicated that the xylanolytic enzyme components of *C. neopsychrotolerans* SL-16 are all inducible, showing significant expression levels of 2.64–24.82 folds upon hemicellulose induction. The genes with the highest expression levels of each GH family (*xyn10A*, *xyn11A* and *xyl43A*) were then selected for functional characterization.

**Table 3:** Genetic information of the 23 xylanolytic genes and *β-tubulin* gene of *C. neopsychrotolerans* SL-16 for qPCR analysis^a^.

Gene	Gene match	DNA (bp)	cDNA (bp)	Protein identity (%)^b^	Relative fold changes^c^
***xyl43A***	**SL-16-605**	**990**	**990**	**84**	**10.57 ± 0.05**
*xyl43B*	SL-16-1949	1,814	1,647	61	9.18 ± 0.10
*xyl43C*	SL-16-4434	1,848	1,848	81	8.33 ± 0.05
*xyl43D*	SL-16-6347	944	891	64	7.51 ± 0.26
*xyl43E*	SL-16-6711	1,699	1,596	68	5.94 ± 0.36
*xyl43F*	SL-16-6818	2,029	1,977	82	5.37 ± 0.21
*xyl43G*	SL-16-7061	1,761	1,761	78	4.67 ± 0.27
*xyl43H*	SL-16-10846	1,344	1,119	80	3.63 ± 0.14
*xyl43I*	SL-16-11780	1,770	1,770	57	2.96 ± 0.04
*xyl43J*	SL-16-11850	1,338	1,338	54	2.95 ± 0.09
***xyn10A***	**SL-16-8247**	**1,224**	**999**	**63**	**24.82 ± 0.16**
*xyn10B*	SL-16-2360	1,114	1,059	71	16.56 ± 0.09
*xyn10C*	SL-16-4503	1,020	1,020	76	13.62 ± 0.10
*xyn10D*	SL-16-7373	1,373	1,257	69	10.75 ± 0.52
*xyn10E*	SL-16-4435	1,446	1,389	68	7.45 ± 0.47
***xyn11A***	**SL-16-9344**	**99**	**696**	**77**	**12.66 ± 0.41**
*xyn11B*	SL-16-72	802	750	86	10.04 ± 0.27
*xyn11C*	SL-16-381	1,124	1,011	59	9.33 ± 0.52
*xyn11D*	SL-16-5111	766	651	74	8.49 ± 0.37
*xyn11E*	SL-16-6019	968	909	71	5.61 ± 0.28
*xyn11F*	SL-16-8056	1,008	879	65	3.76 ± 0.19
*xyn11G*	SL-16-11717	920	780	65	3.27 ± 0.54
*xyn11H*	SL-16-13474	745	639	72	2.64 ± 0.32
*β-tubulin*	SL-16-3619	1,639	1,344	97	1.00 ± 0.07

^a^The gene of each family with the highest mRNA level is shown in bold.

^b^The highest identity to known sequences are shown.

^c^*β-tubulin* was used as the reference gene.

### Production and purification of the recombinant proteins

The gene fragments of *xyn10A*, *xyn11A* and *xyl43A* coding for the mature proteins were subcloned into the *Escherichia coli* BL21(DE3) competent cells for heterologous expression. After 12-h induction with isopropyl β-D-1-thiogalactopyranoside (IPTG) at 15 °C, the *E. coli* cells harboring recombinant plasmids pET-*xyn10A*, pET-*xyn11A* or pET-*xyl43A* were disrupted and showed xylanase or xylosidase activities of 1.87 ± 0.06 U/mL, 1.54 ± 0.04 U/mL and 0.87 ± 0.05 U/mL (pH 6.5, 30 °C and 10 min) with beechwood xylan or *p*-nitrophenyl-β-d-xylopyranoside (*p*NPX) as the substrate, respectively. The crude enzymes were purified to electrophoretic homogeneity by affinity chromatography with a Ni^2+^-NTA chelating agarose column. Each recombinant protein migrated a single band of 23–38 kDa in sodium dodecyl sulfate-polyacrylamide gel electrophoresis (SDS-PAGE, Supplementary Fig. S1), which are essentially identical to their calculated molecular weights (37.6 kDa for Xyl43A, 34.5 kDa for Xyn10A, and 23.5 kDa for Xyn11A).

### Biochemical characterization of the purified recombinant proteins

**Figure 3:**
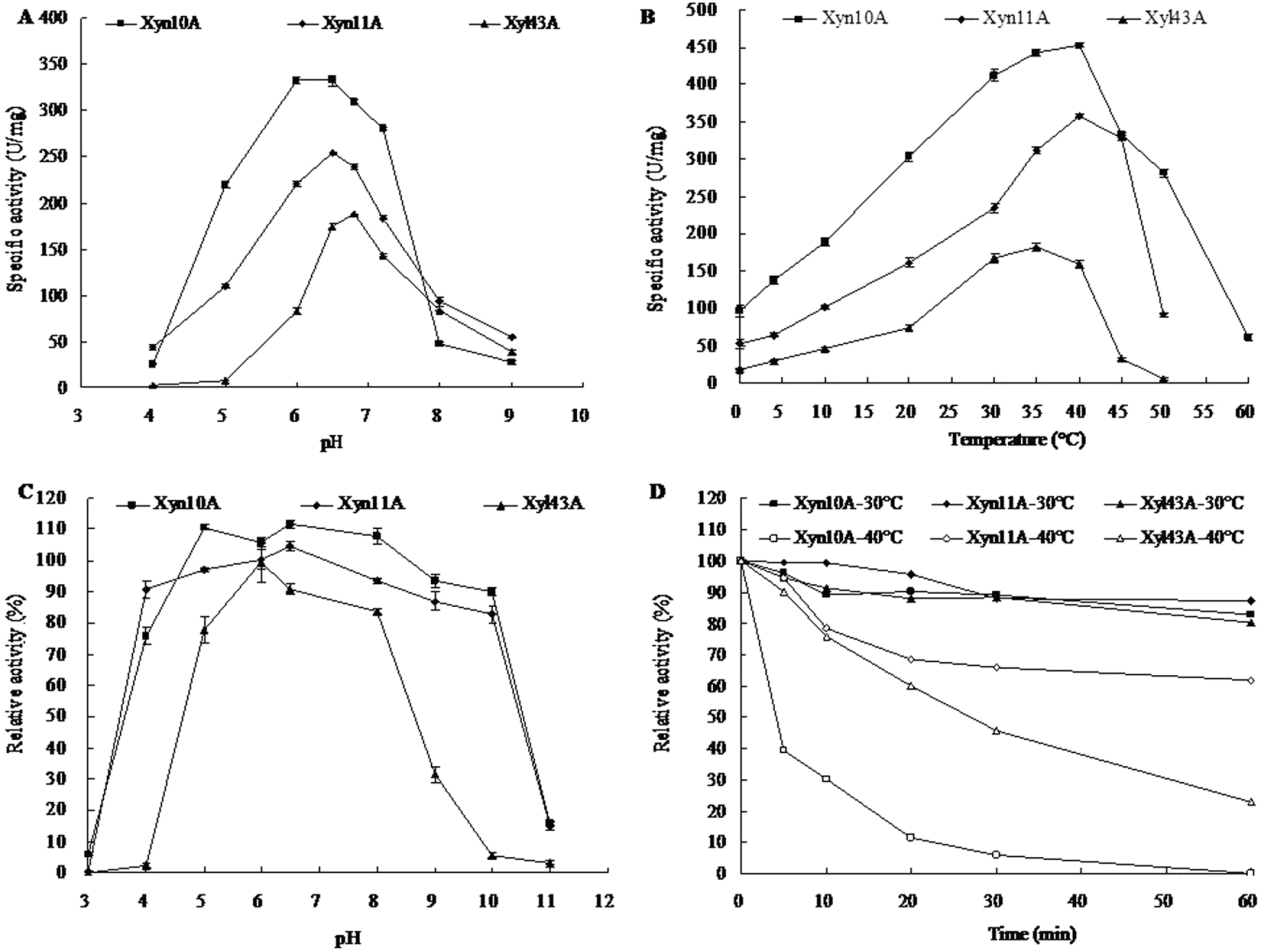
Biochemical characterization of purified recombinant Xyn10A, Xyn101A and Xyl43A from *C. neopsychrotolerans* SL-16. (**A**) pH-activity profiles. (**B**) Temperature-activity profiles. (**C**) pH-stability profiles. (**D**) Temperature-stability profiles. Each value in the panel represents the mean ± SD (n = 3).

The purified recombinant Xyn10A, Xyn11A and Xyl43A exhibited the distinctive features of low-temperature-active enzymes^49^ with similar activity profiles. The enzymes were active over a wide pH range from 4.0 to 9.0 with maxima at neutral pH (6.5–7.0 at 30 °C) (Fig. 3A). In comparison with Xyn10A, Xyn11A and Xyl43A were more alkali-active, remaining 21.4% and 20.6% activity at pH 9.0, respectively. The enzymes had a temperature optimum of 35–40 °C, and remained active even at 0 °C (Fig. 3B). In comparison to Xyn11A and Xyl43A, Xyn10A had higher activity over the whole temperature range tested and retained more activity at 0 °C (21.2% vs. 14.6% and 10.1%). For pH stability, Xyn10A and Xyn11A retained stable (>70% activity) after incubation at room temperature for 1 h over a broad pH range of 4.0 to 10.0 (Fig. 3C). In contrast, the pH stability range of Xyl43A was much narrower. Under similar conditions, it only retained >70% activity at pH 5.0–8.0. The enzymes are much thermolabile, retaining stability at 30 °C after 1 h incubation but losing more than 50% activity at 40 °C within 30 min (Fig. 3D).

The three xylanolytic enzymes were also tested for tolerance against 15 metal ions and chemical reagents at the concentration of 5 mM (Supplementary Table S4). Xyn10A and Xyn11A were tolerant to most chemicals tested (remaining >70% activity), except for Zn^2+^, Pb^2+^, Ag^+^, SDS and EDTA, and their activities were enhanced by Co^2+^, Ni^2+^ or β-mercaptoethanol (>15%). In contrast, the Xyl43A was sensitive to Fe^3+^, Mn^2+^, Cu^2+^, Co^2+^, Ni^2+^, Zn^2+^, Pb^2+^, Ag^+^, SDS, and EDTA, losing 46–100% activity.

### Substrate specificity and xylose tolerance

The substrate spectrum of each enzyme was determined to analyze their degrading capabilities of different carbohydrates (Table 4). Xyn10A had a broader substrate spectrum, including beechwood xylan, birchwood xylan, soluble and insoluble wheat arabinoxylan, Avicel, CMC-Na, lichenin and barley β-glucan. The boarder and shallow active site cleft accessible to more substrates^50^ may account for the wide substrate spectrum of Xyn10A. Xyn11A was observed to be solely active against xylan substrates, including beechwood xylan, birchwood xylan and soluble and insoluble wheat arabinoxylan. For Xyl43A, it had both xylosidase and arabinofuranosidase (against 4-nitrophenyl α-l-arabinofuranoside [pNPAf]) activities. These results indicated that the xylanolytic enzyme system of strain SL-16 is functional against a broad substrate spectrum not limiting to xylan and xylooligosaccharides, which may maximize the utilization of available carbon sources.

**Table 4:** Substrate specificity of Xyn10A, Xyn11A and Xyl43A^a^.

Substrate (concentration)	Relative activity (%)		
	Xyn10A	Xyn11A	Xyl43A
Beechwood xylan (1%)	100.0	100.0	—
Birchwood xylan (1%)	127.4	101.1	—
Soluble wheat arabinoxylan (1%)	116.2	137.2	—
Insoluble wheat arabinoxylan (1%)	39.4	46.2	—
Avicel (1%)	26.0	—	—
CMC (1%)	16.8	—	—
Lichenin (1%)	2.3	—	—
Barley β-glucan (1%)	1.5	—	—
Laminarin (1%)	—	—	—
Filter paper (1%)	—	—	—
*p*NPX (1 mM)	—	—	100.0
*p*NPAf (1 mM)	—	—	69.6
*p*-Nitrophenyl β-d-Glucopyranoside (1 mM)	—	—	—
*p*-Nitrophenyl α-d-Galactopyranoside (1 mM)	—	—	—
*p*-Nitrophenyl α-l-arabinopyranoside (1 mM)	—	—	—
*p*-Nitrophenyl β-d-cellobioside (1 mM)	—	—	—

^a^The specific activities of Xyn10A and Xyn11A towards beechwood xylan (453.0 ± 2.9 U/mg and 357.3 ± 4.4 U/mg, respectively), and Xyl43A to pNPX (181.6 ± 4.6 U/mg) were defined as 100%.

During xylan biodegradation, xylooligosaccharides of various lengths are produced, which would be hydrolyzed into xylose by xylosidase. The inhibitory effect of d-xylose on the xylosidase activity of Xyl43A was tested under the standard conditions in the presence of 0 to 200 mM xylose. The *K*_i_ value of xylose was determined to be 43.3 mM (Supplementary Fig. S2). In comparison to most β-xylosidases, especially those from fungi, that are strongly inhibited by low concentrations of xylose (*K*_i_ for xylose ranging from 2 to 10 mM)^51,52^, Xyl43A with much higher xylose tolerance is particularly interesting for efficient saccharification of xylan.

### Kinetics and thermodynamics

The kinetics of Xyn10A, Xyn11A and Xyl43A were determined at 20 °C and their temperature optima, respectively. In comparison with the kinetics at optimal temperature, Xyn10A retained high *V*_*max*_ and *k*_*cat*_ values but had increased *K*_m_ value at 20 °C, while Xyn11A and Xyl43A had lower *V*_*max*_, *k*_*cat*_, and *K*_m_ values (Table 5). Xyn10A at 20 °C had significantly decreased catalytic efficiency (much lower *k*_*cat*_/*K*_m_ value), the *k*_*cat*_/*K*_m_ value of Xyl43A was comparable to that of optimal conditions, while Xyn11A showed great improvement in catalytic efficiency at lower temperature. It suggested that the three xylanolytic enzymes employed different mechanisms to function at low temperatures.

**Table 5:** Kinetics of Xyn10A, Xyn11A and Xyl43A with beechwood xylan or *p*NPX as the substrate.

Parameters	Xyn10A		Xyn11A		Xyl43A	
	20 °C	40 °C (T_opt_)	20 °C	40 °C (T_opt_)	20 °C	35 °C (T_opt_)
*V*_*max*_ (μmol/min/mg)	322.9 ± 3.1	331.6 ± 2.3	105.3 ± 2.2	298.1 ± 3.4	58.1 ± 1.9	272.4 ± 2.4
*k*_*cat*_ (/s)	185.7 ± 1.7	190.7 ± 1.3	41.2 ± 0.9	116.5 ± 3.3	37.4 ± 0.8	175.2 ± 0.9
*K*_*m*_ (mg/mL)	12.23 ± 1.36	1.87 ± 0.31	0.80 ± 0.06	2.81 ± 0.19	0.69 ± 0.07	2.73 ± 0.37
*k*_*cat*_*/K*_*m*_ (mL/mg · s)	15.2	102.0	51.6	41.5	54.1	67.4

Thermodynamics of the three enzymes were determined by using the differential scanning calorimeter (DSC) instrument. The melting temperature (*T*_*m*_) values of Xyn10A, Xyn11A and Xyl43A were 56.89 °C, 44.03 °C, and 52.55 °C (Supplementary Fig. S3), corresponding to the kinetic results that Xyn11A was more active at low temperature.

### Analysis of the cleavage modes

Hydrolysis products of beechwood xylan and xylooligosaccharides (xylose to xylohextaose) by Xyn10A, Xyn11A and Xyl43A were analyzed by using the high performance anion-exchange chromatography (HPAEC). When using beechwood xylan as the substrate, Xyn10A mainly released xylotriose (67%) and xylobiose (26%), while Xyn11A produced xylotriose (44%), xylotetraose (25%), xylopentaose (13%), and xylobiose (12%). Analysis of the hydrolysis products of xylooligosaccharides confirmed distinctive action modes of Xyn10A and Xyn11A. Xyn10A had no activity against xylobiose and xylotriose, acted weakly on xylotetraose, moderately on xylopentaose, and efficiently on xylohexaose. In contrast, Xyn11A had no capacity of degrading xylobiose, xylotriose and xylotetraose, even lengthening the incubation duration from 30 min to 12 h, and was active against xylopentaose and xylohexaose. The results indicated that Xyn11A requires at least five subsites for binding and cleaves substrate randomly, while Xyn10A requires at least four subsites for binding and cleaves substrate in a relatively strict mode. This difference may also account for the broad functional spectra of Xyn10A, which is more flexible in substrate binding. Xyl43A was capable of degrading all tested xylooligosaccharides and attacking xylan to produce xylose slowly.

### Synergistic actions on xylan degradation

How the three xylanolytic components, xylan-specific Xyn11A, multifunctional Xyn10A, and bifunctional xylosidase-arabinofurasidase Xyl43A, cooperate in conversion of xylan substrate to xylose (pentose) is much intriguing. When using xylans from beechwood, birchwood, and wheat as substrates, the amounts of released reducing sugars (xylose equivalents) by individual or combined enzyme(s), each at the dosage of 20 U/g dry matter, were determined. As shown in Table 6, Xyn10A, Xyn11A and Xyl43A alone released 1.74–3.82 mM, 1.22–2.49 mM and 0.15–0.33 mM of reducing sugars from xylan substrates, respectively. When treated xylan substrates with simultaneous enzyme combination, 2.71–4.79 mM of reducing sugars were released, in which xylose accounted for 1.94–2.79 mM and the xylose conversion rates (XCRs) reached up to 71.5%. Considering the spatial and temporal relationships of these enzymes, sequential instead of simultaneous combination might be much closer to the fact that extracellular Xyn10A and/or Xyn11A first cleaves the xylan to release xylooligosaccharides, which are further transported into cellular compartment for Xyl43A hydrolysis. Thus sequential enzyme combinations with Xyl43A as the last step were also used to degrade xylan substrates. When added the enzymes at the order of Xyn11A + Xyn10A → Xyl43A, Xyn10A → Xyn11A → Xyl43A, or Xyn11A → Xyn10A → Xyl43A, 3.29–4.98 mM of reducing sugars were released. Xylose as the only detected xylooligosaccharide consisted of 2.80–3.83 mM of the reducing sugars, with the XCRs of 72.3–91.0%. In comparison to the sum of reducing sugars released by individual enzymes, no significant synergistic effect was observed in the enzyme combinations. In contrast, significant synergy was observed in xylose production. When the enzymes combined, more xylose (1.94–3.83 mM vs. 0.25–0.48 mM) was released, and the synergy was up to 15.3-fold. It suggested that Xyn10A and Xyn11A may have both collaboration and competition in xylan hydrolysis, and a sequential strategy with Xynl43A at the last step is of great efficiency in xylan conversion.

**Table 6:** Xylan-degrading performance of simultaneous and sequential combinations of Xyn11A, Xyn10A and Xyl43A^a^.

Enzyme(s)	Beechwood xylan			Birchwood xylan			Soluble wheat arabinoxylan		
	Reducing sugars (mM)	Xylose (mM)	XCR (%)	Reducing sugars (mM)	Xylose (mM)	XCR (%)	Reducing sugars (mM)	Xylose (mM)	XCR (%)
Xyn10A	1.74 ± 0.09	0.18 ± 0.02	10.3	3.25 ± 0.06	0.16 ± 0.01	4.9	3.82 ± 0.16	0.35 ± 0.02	9.1
Xyn11A	1.22 ± 0.04	0.03 ± 0.00	2.4	2.48 ± 0.09	0	—	2.49 ± 0.09	0	—
Xyl43A	0.33 ± 0.02	0.27 ± 0.02	81.8	0.15 ± 0.03	0.09 ± 0.03	60.0	0.15 ± 0.02	0.09 ± 0.00	60.0
Sum of Xyn10A, Xyn11A and Xyl43A	3.29	0.48	14.6	5.88	0.25	4.3	6.46	0.44	6.8
Xyn10A + Xyn11A + Xyl43A	2.71 ± 0.07^a^	1.94 ± 0.08^a^	71.5	3.97 ± 0.04^a^	2.68 ± 0.10^a^	67.5	4.79 ± 0.09a	2.79 ± 0.04a	58.2
Xyn10A + Xyn11A → Xyl43A	3.37 ± 0.13^b^	2.75 ± 0.10^b^	81.6	3.87 ± 0.14^a^	2.88 ± 0.07^a^	74.4	4.34 ± 0.06b	2.44 ± 0.03b	56.2
Xyn10A → Xyn11A → Xyl43A	3.44 ± 0.12^b^	2.80 ± 0.05^b^	81.5	4.23 ± 0.13^b^	3.78 ± 0.08^b^	89.4	4.80 ± 0.14a	3.25 ± 0.08c	67.7
Xyn11A → Xyn10A → Xyl43A	3.29 ± 0.09^b^	2.92 ± 0.09^b^	88.7	4.21 ± 0.11^b^	3.83 ± 0.09^b^	91.0	4.98 ± 0.12 a	3.60 ± 0.07d	72.3

^a^The compositions of beechwood xylan, birchwood xylan and soluble wheat arabinoxylan are: xylose/arabinose ratio of ~90:10, xylose/arabinose ratio of ~90:10, and xylose/glucose/arabinose ratio of ~75:15:10, respectively.

^b^The amounts of reducing sugars (xylose equivalents) were determined by using the DNS method; *significant difference at *p* < 0.05 (Tukey’s test by Origin Pro 8).

^c^The amounts of xylose were determined by using the HPLC.

^d^XCR, the xylose conversion rate, which is defined as the conversion rate of reducing sugars to xylose.

### Analysis of the cold-adapted mechanisms

The parameters related to structure flexibility of Xyn10A, Xyn11A and Xyl43A and their structure-resolved counterparts were analyzed and shown in Table 7. The GH10 xylanase of *Thermoascus aurantiacus* (*Ta*Xyn10)^53,54^ and GH11 xylanase of *Thermomyces lanuginosus* (*Tl*Xyn11)^55^ are thermophilic, while the GH43 xylosidase-arabinofuranosidase from a compost metagenome (CoXyl43)^56,57^ is mesophilic. Their structure elements, protein interaction and accessible surface area were compared to reveal the cold-adapted strategies. In comparison to the mesophilic and thermophilic counterparts, the three enzymes have less hydrophobic residues in common. Moreover, Xyn10A has more Gly and higher Gly/Pro ratio, Xyn11A has more Arg and higher Gly/Pro ratio, and Xyl43A has more Gly. Protein interactions, including hydrogen bonds, salt bridge, disulfide bonds, hydrophobic bonds, aromatic-aromatic interactions, and cation-Pi interactions, are also negatively correlated with enzyme flexibility. Relative to the counterparts, Xyn10A has less hydrogen and hydrophobic bonds and no disulfide bond, Xyn11A has less salt bridges and cation-Pi interactions and no disulfide bond, and the Xyl43A has less protein interactions checked in this study. Another structure feature of enzyme flexibility is increased interactions with the solvent. Xyn10A and Xyn11A have larger accessible surface area (ASA) but vary in the amounts of exposed nonpolar, polar and charged ASA, while Xyl43A has less ASA. It suggested that the three xylanolytic enzymes employ different strategies to function at low temperatures.

**Table 7:** Comparison of the parameters affecting enzyme flexibility of Xyn10A, Xyn11A and Xyl43A and their thermophilic and mesophilic homologues.

Parameters	Xyn10A	*Ta*Xyn10 (2BNJ)	Xyn11A	*Tl*Xyn11 (1YNA)	Xyl43A	CoXyl43 (5GLK)
Temperature optimum (°C)	40	70–75	40	60	35	55
Number of amino acids	333	329	232	225	330	369
Sequence identity (%)	63		67		62	
**Amino acid composition** ^**a**^						
Hydrophobic residues (%)	38.44	39.82	28.02	31.56	29.09	31.71
No. of glycine residue (%)	23 (6.91)	22 (6.69)	29 (12.5)	32 (14.2)	25 (7.58)	34 (9.21)
No. of Arginine residues (%)	9 (2.70)	12 (3.65)	12 (5.17)	9 (4.00)	11 (3.33)	10 (2.71)
No. of Proline residues (%)	10 (3.00)	17 (5.17)	7 (3.02)	8 (3.56)	22 (6.67)	24 (6.50)
Gly/Pro ratio	2.30	1.29	4.14	4.00	1.14	1.42
**Protein interaction** ^**b**^						
No. of hydrogen bonds	632	691	365	311	184	935
No. of salt bridge	26	25	13	18	8	86
No. of disulfide bridges	0	1 (C255-C261)	0	1 (C110-C154)	0	0
No. of hydrophobic bonds	247	297	141	127	101	600
No. of aromatic-aromatic interactions	13	11	21	20	8	55
No. of Cation-Pi interactions	8	7	3	6	4	21
**Accessible surface area (ASA)** ^**c**^						
Total ASA (Å^2^)	11637.4	10823.0	8106.3	8032.7	8777.0	25010.1
Exposed nonpolar ASA (Å^2^)	6550.6	6214.2	3819.9	4345.2	5418.3	13826.0
Exposed polar ASA (Å^2^)	3498.5	3477.2	3731.6	2477.4	1857.9	5170.2
Exposed charged ASA (Å^2^)	1588.3	1131.6	554.7	1210.1	1500.7	6023.9

^a^Vector NTI Advance v10.0 was used to analyze the composition of amino acids.

^b^PIC (http://pic.mbu.iisc.ernet.in/) was used to calculate the interprotein interactions.

^c^ADAR v1.8 (http://vadar.wishartlab.com/) was used to calculated the accessible surface area of protein.

## Discussion

The genus *Cladosporium* is one of the largest and most heterogeneous genera of hyphomycetes that are widely distributed in all types of environments^27,28,58,59^. It has been attracting much research interest in recent years because of the broad physiological capabilities (i.e. psychroolerance, osmotolerance, halotolerance, radiotolerance, and thermotolerance)^15,17,21,25,60,61^ and pathogenicity in plants, animals and humans^62,63^. Two genomes of *Cladosporium* have been sequenced and functionally annotated, and their nutritional modes may account for the evolution of functional genes. The tomato leaf mold pathogen *C. fulvum* has a genome of 61.1 Mb that contains 14,127 predicted genes^31^, while the human allergen and occasional pathogen *C. sphaerospermum* has a genome of 26.9 Mb that contains 9,652 predicted genes^36,64^. Both genomes encode wide arrays of CAZymes (445 for *C. fulvum* and 605 for *C. sphaerospermum*), which are related to their nutritional strategy and host specificity^45,65^. In comparison to the biotrophic *C. fulvum* and saprophytic *C. sphaerospermum*, the hemibiotrophic *C. neopsychrotolerans* SL-16 isolated from the rhizosphere soil of snow lotus has a moderate-size genome (35.9 Mb) that contains 13,456 predicted genes and encodes 571 CAZymes. Gene classification analysis indicated that most of the predicted genes of *Cladosporium* are related to the transport and metabolism of carbohydrate, protein and lipid. In comparison to other known biomass-degrading fungal strains, all the three *Cladosporium* species tend to have more CAZymes (58–92 vs. 31–53) to degrade hemicellulose (Supplementary Table S2). We thus infer that efficient utilization of hemicellulose is a key physiological character of *Cladosporium*.

*C. neopsychrotolerans* is eurypsychrophilic that grows well in cold environments. To undermine its cold-active hemicellulose-utilizing mechanism, we selected a xylanolytic enzyme system, which is supposed to act cooperatively to convert xylan into its constituent sugars. Upon xylan induction at low temperature (15 °C), the 23 xylanolytic enzymes showed high expression levels (2.64–24.82 folds, Table 3), which agree to the conclusion that all xylanolytic genes are inducible^66^. Xylanolytic enzymes from fungi generally exhibit great activity under acidic and mesophilic conditions^67^. In contrast, those from erypsychrophilic fungi are mostly neutral and cold-active, and remain active at alkaline pH and even 0 °C^18,68–70^. The three xylanolytic enzymes from *C. neopsychrotolerans* SL-16 share similar cold-adaptive properties, including maximal activity at neutral (pH 6.5) and low temperature (35–40 °C) and inactivation by the application of moderate heat (>40 °C). In comparison to the two cold-active fungal xylanases from psychrotrophic *Penicillium chrysogenum* and *Bispora antennata*^68,69^ that have temperature optima at 25 °C and 35 °C, the xylanolytic enzymes of eurypsychrophilic *C. neopsychrotolerans* showed similar or higher temperature optima and retained less or comparable activity at 0 °C (10.1–21.2% vs. 50% and 20%). Further kinetic analysis of these enzymes revealed different cold-adaptive catalytic mechanisms: under low temperature (20 °C), Xyn10A retained high enzyme velocity and turnover number, while Xyn11A and Xyl43A showed increased substrate affinity (Table 5). As results, Xyn10A showed higher catalytic efficiency (102.0 vs. 15.2 mL/mg·s) at optimal temperature (40 °C) than at 20 °C, while Xyn11A and Xyl43A showed higher or comparative catalytic efficiencies (51.6 and 41.5 mL/mg·s and 54.1 and 67.4 mL/mg·s, respectively) at 20 °C. In combination with their *T*_*m*_ values (44.03–56.89 °C), Xyn11A has psychrophilic nature while Xyn10A and Xyl43A are psychrotolerant. Moreover, these enzymes also varied in pH adaptability and stability and chemical resistance. In comparison with Xyn10A and Xyn11A, Xyl43A had adaptability and stability over a narrower pH range and was sensitive to most tested metal ions and chemical reagents. This divergence might be ascribed to their evolution of different functional strategies: Xyn10A and Xyn11A are extracellular and more adaptive to harsh conditions, while intracellular Xyl43A is relatively demanding and lacks adaptation to environmental stress. The similar and complementary properties of these xylanolytic enzymes may contribute to the efficient hemicellulose utilization of *C. neopsychrotolerans* SL-16 over a broad temperature range (0–60 °C).

Xylanases of GH11 are “true xylanases” that have no non-xylanase activities, while xylanolytic enzymes of GH10 and GH43 have broad substrate specificities^71^. The functionally characterized xylanolytic enzymes of strain SL-16 showed the same substrate spectra as described above. Xyn10A had broad substrate specificity including xylan, cellulose and glucan, Xyn11A was specific for xylan substrates, and Xyl43A had both xylosidase and arabinofuranosidase activities. Combination of these enzymes would broaden the substrate spectrum and act synergistically on complex biomass by using different cleavage modes^67,72^. In this study, the hydrolysis capacities of the xylanolytic enzyme system against three xylan substrates were tested. Of them, hardwood xylan (beechwood and birchwood) contains approximately 90% xylose that are linked by β-1,4-glycosidic bonds as the main chain, while wheat arabinoxylan is featured with relatively complex composition (xylose/glucose/arabinose of ~75:15:10)^73,74^. Although all enzyme combinations had no significant synergy (0.74–1.04-fold) on the release of reducing sugars, more reducing sugars (up to 1.27-fold) were detected in the sequential rather than simultaneous reactions with hardwood xylan as the substrate (Table 6). Considering the cleavage modes of Xyn10A and Xyn11A that require at least four and five subsites respectively, we conjecture that these two xylanases may have collaboration as well as competition in xylan hydrolysis. From the point of view of xylose production, the enzyme combinations acted synergistically on the xylose release (6.08–15.32-fold) with XCRs of 58.2–91.0%. Moreover, the XCRs of sequential enzyme combination Xyn11A → Xyn10A → Xyl43A are close to the xylose contents of the three tested substrates (75% and 90%). It indicated that the xylanolytic enzyme system of *C. neopsychrotolerans* SL-16 with a broad substrate spectrum is highly efficient in the hydrolysis of different xylan substrates, even under harsh environmental conditions.

In contrast to the extensively studied thermostable xylanases, limited information is available for the psychrophilic xylanases. To our best knowledge, there are eleven functionally characterized cold-active xylanases of GH10 and GH11 including two fungal ones^69,70^, one cold-active bacterial xylanase of GH10 with structure resolved^75^, and no cold-active GH43 enzyme. Thus Xyl43A is the first characterized cold-active xylosidase-arabinofurasidase known so far. It’s commonly acknowledged that cold-adapted enzymes have increased structure flexibility at the expense of stability to achieve efficient hydrolysis at low temperature^49^. The enzyme flexibility is characterized by decreased contents of Arg and Pro reduced interactions, and increased Gly content, Gly/Pro ratio, hydrophobic accessible area^9–11,76^. To identify the cold-active catalytic mechanism of the xylanolytic enzyme system of *C. neopsychrotolerans* SL-16, three thermophilic or mesophilic counterparts *Ta*Xyn10^53,54^, *Tl*Xyn11^55^, and CoXyl43^56,57^ were selected for comparison of some structural features (Table 7). Of the three xylanolytic enzymes, only Xyn10A followed the cold-adapted strategy that high Gly content, increased Gly/Pro ratio and surface hydrophobicity, and less Arg and Pro contribute to enzyme flexibility^11^. Of intraprotein interactions related to enzyme stability, Xyn10A and Xyn11A each possessing one disulfide bridge have less hydrogen bonds and less salt bridges, respectively, while Xyn43A without disulfide bridge has less hydrogen bonds and salt bridges instead. Taken together, we conclude that the cold-adaptation feature of the xylanolytic enzyme system of *C. neopsychrotolerans* SL-16 is a synergistic effect of different factors, and intracellular and extracellular proteins may employ different strategies to function at low temperatures.

## Methods

### Fungal strain

*C. neopsychrotolerans* SL-16 was isolated from alpine soil of the Tianshan Mountain (46°06′N, 86°50′E)^23^. To test the ability of SL-16 to grow at different temperatures, it was grown in wheat bran medium^43^ at 4–30 °C for 7 days. The lignocellulose-degrading capability of the fungus were assayed at pH 6.0 and 30 °C for 10 with 1% (w/v) beechwood xylan, barley β-glucan, and carboxymethyl cellulose (CMC) as substrates as described below.

### Genomic analysis

The genomic DNA of *C. neopsychrotolerans* SL-16 was extracted using the CTAB method. The 500-bp insert library was sequenced using the Illumina PE250 system. The clean reads were then assembled by SOAPdenovo (http://soap.genomics.org.cn, v2.04)^77^ for data processing. Genes were subsequently predicted using Augustus 3.2.1^78^ and GeneMark-ES 4.21^79^ with default parameters, which were further integrated by the GLEAN (https://sourceforge.net/projects/glean-gene/). All predicted gene models were functionally annotated against InterProScan^80^, SwissProt^81^, KEGG^82^, and KOG^83^. Interpro and SwissProt hits were used to map GO terms (www.geneontology.org), and KEGG hits were used to assign EC numbers (www.expasy.org/enzyme). Genes associated with carbohydrate utilization were identified by performing a local BLASTp against the CAZy database (http://www.cazy.org) using the criteria of e-value threshold ≤1e^−5^, identity exceeding 50% and subject coverage exceeding 70%. SignalP^84^ and TMHMM^85^ were employed to predict the presence of signal peptide and transmembrane α-helix structures. The protein sequences of eight biomass-degrading fungal representatives were downloaded from different databases for orthologous gene comparison (Table 2).

### RNA extraction and RT-PCR of xylanolytic genes

The mycelia of strain SL-16 were collected after 2-day-growth in wheat bran medium at 15 °C and immediately ground to a fine powder in liquid nitrogen. Total RNA was extracted using the SV Total RNA Isolation System (Promega) according to the manufacturer’s protocol. The quantity and purity of RNA was determined using an Ultrospec 2100 pro UV/visible spectrophotometer (Amersham Biosciences) based on the absorbance ratios of A_260_/A_280_ and A_260_/A_230_. cDNA was synthesized according to the protocol of ReverTra Ace-α-TM kit (TOYOBO). The gene fragments coding for mature xylan-main-chain degrading enzymes (xylanase and xylosidase) with >50% identity to known proteins were amplified by PCR and ligated into the pEasy-T3 vector (Tiangen) for Sanger sequencing.

### qPCR of the xylanolytic genes

qPCR was performed using the QuantStudio 6 Flex real-time qPCR system (Thermo Fisher Scientific) with the SYBR Green SuperReal PreMix (TianGen), SL-16 cDNA template, and specific primers of each gene (Supplementary Table S5). Standard curves were generated with 10–10^7^ copies of each gene. All qPCR amplifications followed the thermal program consisting of the following cycles: 95 °C for 5 min, 40 cycles of 94 °C for 30 s, 60 °C for 30 s, and 72 °C for 30 s. Fluorescence data were collected at the last step of each cycle. The relative fold change of each gene was calculated from the *C*_*t*_ values after normalization against *β-tubulin*. All operations for qPCR followed the MIQE^86^.

### Production and purification of recombinant xylanolytic enzymes

The gene fragments of *xyn10A*, *xyn11A* and *xyl43A* with highest expression levels of each family and vector pET-30a(+) (Novagen) were digested by restriction enzymes (*Nde*I, *Not*I or *Eco*RI), purified, and connected by the T4 DNA ligase (New England Biolab) to construct recombinant plasmids, which were further transformed into *E. coli* BL21(DE3) competent cells. Enzyme induction with IPTG (0.6–0.8 mM) at 15 °C and purification through nickel affinity chromatography followed the previous protocols^87^. Xylanolytic activities of the sonication-disrupted cell pellets and purified recombinant enzymes were assayed as described below. The molecular mass and purity of each purified recombinant enzymes were evaluated by the SDS-PAGE. The protein concentrations were determined using bovine serum albumin as the standard^88^.

### Biochemical characterization

Xylanase, mannanase, CMCase or glucanase activity was assayed using 3,5-dinitrosalicylic acid (DNS) method^89^ with 1% (w/v) beechwood xylan, locust bean gum, CMC or barley β-glucan (Sigma-Aldrich) as the substrate. One unit (U) of xylanase/mannanase/CMCase/glucanase activity was defined by the amount of enzyme releasing 1 μmol of reducing sugar per min at given assay conditions. The xylosidase activity towards *p*NPX (1 mM) was assayed as described by Shao *et al*.^90^. One unit of xylosidase activity was defined as the amount of enzyme that released 1 μmol of *p*-nitrophenol per min.

The pH properties of Xyn10A, Xyn11A and Xyl43A were determined in the 100 mM of McIlvaine buffer (pH 3.0–8.0), Tris-HCl (pH 8.0–9.0) and glycine-NaOH (pH 9.0–12.0). The pH-activity profiles were examined at 30 °C over the pH range of 3.0–9.0 for 10 min. The temperature optima were examined at each optimal pH over the temperature range of 0 to 60 °C. For pH stability assays, all enzymes were pre-incubated at pH 3.0–11.0, 30 °C for 1 h without the substrate, and their residual enzyme activities were determined under optimal conditions. The thermostability assays were performed by incubating the enzymes at optimal pH and at 30 °C or 40 °C without substrate for 0–60 min, and then measuring the residual enzyme activities under the optimal assay conditions.

The effects of different metal ions, chemical reagents and detergents on enzyme activities were determined under optimal conditions and compared to the blank controls.

### Substrate specificity and xylose tolerance

The substrate specificities of Xyn10A, Xyn11A and Xyl43A were tested by measuring their enzyme activities against 1% (w/v) of polysaccharides (birchwood xylan, beechwood xylan, soluble/insoluble wheat arabinoxylan, sugar beet arabinan, debranched AZCL-arabinan, barley β-glucan, laminarin, lichenin, CMC-Na, and Avicel) or 1 mM of *p*NP derivatives including *p*NPX, *p*NPAf, *p*NPG, *p*NPGal, *p*NPAb, and *p*NPC. All substrates were purchased from Sigma-Aldrich or Megazyme.

The inhibition constant (*K*_i_) of d-xylose for Xyl43A was determined by fitting to the Dixon plot^91^. Reaction systems (500 μL) containing 125 μL of 1.0 or 1.25 mM *p*NPX, 275 μL of d-xylose solution (0–400 mM in McIlvaine buffer, pH 6.8) and 100 μL of suitably diluted enzyme were incubated at room temperature for 10 min. Enzyme activities were determined as described above.

### Kinetics and thermodynamics

The *K*_m_ and *V*_max_ values of Xyn10A, Xyn11A and Xyl43A at optimal pH and 20 °C or optimal temperature were determined by using 1–10 mg/mL beechwood xylan or 1–10 mM *p*NPX as the substrate. The GraphPad Prism v5.01 (La Jolla) was used for data analysis using the Michaelis-Menten model. DSC was used to assay the thermodynamics of Xyn10A, Xyn11A and Xyl43A on a MicroCal™ VP-Capillary DSC (GE Healthcare) as described previously^92^. Aliquots of proteins (200 μg) were dissolved in 1 mL of 20 mM McIlvaine buffer (pH 6.5) and loaded into the capillary automatically. The *T*_*m*_ value corresponded to the maximum of the transition peak over 20–90 °C within a heating and scanning rate of 2 °C/min. The same McIlvaine buffer was used as the blank control.

### Synergistic actions of Xyn10A, Xyn11A and Xyl43A on xylan degradation

Reaction systems (1 mL) containing 0.5 U of Xyn10A, Xyn11A or Xyl43A and 1% (w/v) substrate (beechwood xylan, birchwood xylan or soluble wheat arabinoxylan) in McIlvaine buffer (pH 6.5) were incubated at room temperature for 12 h and treated as baselines. The simultaneous reactions of Xyn10A, Xyn11A and Xyl43A contained 0.5 U of each enzyme. For sequential reactions, first reactions containing either Xyn10A or Xyn11A (0.5 U of each) were incubated at room temperature for 12 h, boiled in water bath for 10 min to inactivate the enzyme(s), followed by secondary and tertiary incubation(s) with the other xylanase and xylosidase for another 12 h. The released reducing sugars in the supernatants were measured as xylose equivalents by using the DNS method, and the appropriately diluted hydrolysates (50–200 times) were analyzed by the HPAEC (model 2500, Dionex) equipped with a pulsed amperometric detector (PAD) and xylooligosaccharides (Sigma-Aldrich) as standards. The XCR was defined as the percentage of xylose amount against the total amount of reducing sugars (xylose equivalents) released. The degree of synergy was defined as the ratio of xylose released when enzymes were incubated simultaneously or sequentially to the sum of the xylose released by each enzyme alone^93^.

### Cold-adapted strategies of Xyn10A, Xyn11A and Xyl43A

Three structure-resolved thermophilic or mesophilic homologues, the GH10 xylanase of *T. aurantiacus* (*Ta*Xyn10; pdb: 2BNJ)^53^, GH11 xylanase of *T. lanuginosus* (*Tl*Xyn11; pdb: 1YNA)^55^, and GH43 xylosidase-arabinofuranosidase of a metagenome (*Co*Xyl43; pdb: 5GLK)^57^, were selected for the analysis of cold-adapted strategies. Homology modeling of Xyn10A, Xyn11A and Xyl43A was conducted by using the Swiss-modeler (https://swissmodel.expasy.org/interactive). The amino acid composition was analyzed by the Vector NTI Advance v10.0 software. PIC (http://pic.mbu.iisc.ernet.in/) and VADAR v1.8 (http://vadar.wishartlab.com/) were used to calculate the protein interactions and ASA.

## Electronic supplementary material

Supplementary materials
