# Supplementary material for: Insight into the cold adaptation and hemicellulose utilization of Cladosporium neopsychrotolerans from genome analysis and biochemical characterization
Source: Sci Rep. 2018 Apr 17;8:6075. doi: 10.1038/s41598-018-24443-7 (PMC5904165; doi:10.1038/s41598-018-24443-7)
Supplement: Supplementary file 1 — Supplementary materials [file 41598_2018_24443_MOESM1_ESM.pdf]

Supplementary materials to

Insight into the cold adaptation and hemicellulose utilization of *Cladosporium neopsychrotolerans* from genome analysis and biochemical characterization

Rui Ma [1,2], Huoqing Huang [1], Yingguo Bai [1], Huiying Luo [1], Yunliu Fan [2] & Bin Yao [1*]

[1] Key Laboratory for Feed Biotechnology of the Ministry of Agriculture, Feed Research Institute, Chinese Academy of Agricultural Sciences, Beijing, China. [2] Biotechnology Institute, Chinese Academy of Agricultural Sciences, Beijing, China

Keywords: *Cladosporium neopsychrotolerans*; Carbohydrate Active enZyme (CAZyme); cold adaptation; genome; xylanolytic genes

***Correspondence:**

Bin Yao, Key Laboratory for Feed Biotechnology of the Ministry of Agriculture, Feed Research Institute, Chinese Academy of Agricultural Sciences, Beijing 100081, China

Fax: 86 10 82106053; Tel.: 86 10 82106054; E-mail: binyao@caas.cn; yaobin@caas.cn

Table S1. CAZyme families predicted in the genomes of *C. neopsychrotolerans* SL-16, *C. fulvum* CBS13901 and *C. sphaerospermum* UM843.[a]

AA				CBM				CE				GH								GT				PL			
Family	Cn	Cf	Cs	Family	Cn	Cf	Cs	Family	Cn	Cf	Cs	Family	Cn	Cf	Cs	Family	Cn	Cf	Cs	Family	Cn	Cf	Cs	Family	Cn	Cf	Cs
AA1	16	-	10	CBM1	24	0	12	CE1	5	5	32	GH1	7	3	4	GH54	1	1	1	GT1	12	6	8	PL1	11	3	8
AA2	4	-	7	CBM2	3	0	0	CE2	1	0	1	GH2	8	5	8	GH55	2	5	3	GT2	1	18	17	PL3	6	3	4
AA3	28	-	20	CBM6	1	0	0	CE3	3	3	1	GH3	29	19	19	GH61	0	2	0	GT3	1	1	1	PL4	1	2	0
AA4	1	-	1	CBM13	6	1	0	CE4	2	3	6	GH5	22	16	17	GH62	3	0	3	GT4	0	5	5	PL9	1	2	1
AA5	1	-	1	CBM14	1	1	2	CE5	14	11	8	GH6	1	0	1	GH63	1	1	2	GT5	3	2	1	PL7	0	1	0
AA6	1	-	1	CBM18	4	7	2	CE7	0	0	2	GH7	2	2	2	GH64	4	5	5	GT8	7	10	7	PL22	0	0	1
AA7	2	-	22	CBM19	1	0	3	CE8	3	2	7	GH10	5	2	4	GH65	1	1	1	GT15	3	3	3				
AA8	2	-	4	CBM20	10	4	5	CE9	2	2	2	GH11	8	2	5	GH67	1	1	1	GT17	1	3	0				
AA9	14	-	10	CBM21	1	1	1	CE10	0	0	50	GH12	4	4	1	GH71	1	2	1	GT20	3	3	3				
AA10	2	-	1	CBM27	0	0	1	CE12	2	2	2	GH13	9	15	16	GH72	7	8	6	GT21	1	1	1				
AA11	1	-	0	CBM32	0	1	2	CE14	0	0	1	GH15	4	2	2	GH74	0	0	2	GT22	4	4	4				
AA12	1	-	0	CBM35	4	2	2	CE16	2	7	2	GH16	14	16	12	GH75	1	1	0	GT24	1	1	1				
				CBM42	1	1	1					GH17	6	8	7	GH76	11	8	9	GT25	5	2	4				
				CBM43	2	1	1					GH18	13	13	6	GH78	6	6	3	GT26	0	0	1				
				CBM48	4	3	1					GH20	2	2	1	GH79	2	6	1	GT28	0	0	1				
				CBM50	0	4	2					GH25	0	0	1	GH81	1	1	1	GT30	1	0	0				
				CBM52	0	1	1					GH27	4	1	4	GH88	2	2	2	GT31	7	9	4				
				CBM61	0	0	1					GH28	13	15	10	GH92	4	7	4	GT32	5	5	5				

				CBM63	2	1	2					GH29	2	1	1	GH93	4	2	3	GT33	1	1	1				
				CBM67	0	0	2					GH30	4	2	3	GH95	3	2	1	GT34	2	6	3				
												GH31	11	15	11	GH99	0	0	1	GT35	1	1	1				
												GH32	6	4	5	GH105	3	4	4	GT39	3	3	3				
												GH33	1	0	0	GH106	0	0	1	GT41	1	1	0				
												GH35	6	6	4	GH109	0	0	12	GT48	1	1	1				
												GH36	2	2	1	GH114	1	1	1	GT50	1	1	1				
												GH37	3	2	2	GH115	1	1	3	GT57	2	2	3				
												GH38	1	1	1	GH121	0	0	1	GT58	1	1	1				
												GH39	2	2	0	GH125	2	3	2	GT59	1	0	1				
												GH42	1	1	1	GH127	1	1	0	GT62	3	3	3				
												GH43	24	22	18	GH128	2	5	3	GT66	1	1	1				
												GH45	3	1	1	GH130	1	0	1	GT69	1	2	3				
												GH47	6	8	6	GH131	2	0	2	GT71	2	4	1				
												GH51	2	2	3	GH132	2	0	2	GT76	1	0	1				
												GH53	2	2	2	GH133	1	0	0	GT90	6	5	8				
Total	73	-	77	Total	64	28	41	Total	34	35	114					Total	298	268	261	Total	83	105	98	Total	19	9	14

[a]AA, auxiliary activities; CBM, carbohydrate-binding modules; CE, carbohydrate esterases; GH, glycoside hydrolases; GT, glycosyltransferases; PL, polysaccharide lyases; Cn, *C. neopsychrotolerans* SL-16; Cf, *C. fulvum* CBS13901; Cs, *C. shaerospermum* UM843.

Table S2. Numbers of CAZymes of *Cladosporium* spp. and eight biomass-degrading fungi involved in the degradation of plant cell wall.[a]

**Strain**	**Hemicellulose**	**Pectin**	**Hemicellulose /Pectin side-chain**	**Cellulose**	**CBM**	**Total**
C. neopsychrotolerans	**92**	**71**	**55**	8	9	**235**
C. fulvum	58	40	31	9	6	144
C. sphaerospermum	78	39	32	7	17	173
A. fumigatus	43	44	29	10	27	153
A. nidulans	47	48	29	9	18	151
C. globosum	53	22	31	**19**	**65**	190
M. thermophila	37	15	23	14	36	125
N. crassa	31	9	13	15	34	102
P. chrysogenum	37	23	23	8	12	103
T. reesei	35	15	21	15	42	128
T. terrestris	32	11	6	10	18	77

[a] The functions of CAZymes in hydrolysis of cellulose, hemicellulose, pectin, side chains of hemicellulose and pectin, and carbohydrate binding were predicted according to Amselem *et al.* [44]. The highest value of each column is shown in bold.

Table S3. Standard curves of the 23 xylanolytic genes and *β-tubulin* gene of *C. neopsychrotolerans* SL-16 for qPCR analysis.

Gene	Curve fit equation	R^2	PCR efficiency (%)
xyl43A	Y= −3.265X + 0.026	0.994	102. 4
xyl43B	Y= −3.403X + 4.113	0.996	96.7
xyl43C	Y= −3.178X − 1.426	0.990	101. 3
xyl43D	Y= −3.297X + 3.562	0.997	98.9
xyl43E	Y= −3.336X + 0.687	0.993	99. 2
xyl43F	Y= −3.498X − 2.134	0.993	97.9
xyl43G	Y= −3.116X + 1.078	0.999	102. 4
xyl43H	Y= −3.267X − 1.913	0.995	98.1
xyl43I	Y= −3.008X − 2.718	0.998	99. 4
xyl43J	Y= −3.369X − 2.005	0.991	98.7
xyn10A	Y= −3.362X + 0.047	0.998	98.3
xyn10B	Y= −3.414X− 1.939	0.992	96.9
xyn10C	Y= −3.314X + 3.202	0.999	100.4
xyn10D	Y= −3.362X + 0.047	0.997	98.3
xyn10E	Y= −3.415X − 1.065	0.993	106.5
xyn11A	Y= −3.175X − 1.191	0.995	97.9
xyn11B	Y= −3.096X + 0.118	0.991	99.5
xyn11C	Y= −3.428X + 1.423	0.994	101.5
xyn11D	Y= −3.540X − 2.584	0.990	98.5

xyn11E	Y= −3.099X + 1.336	0.994	97.7
xyn11F	Y= −3.257X + 1.080	0.998	103.7
xyn11G	Y= −3.435X − 3.002	0.991	99.5
xyn11H	Y= −3.112X + 0.913	0.997	98.7
β-tubulin	Y= −3.216X + 1.454	0.995	104.7

Table S4. Effects of metal ions and chemical reagents on the activities of Xyn10A, Xyn11A and Xyl43A. [a]

Chemicals	Xyn10A	Xyn11A	Xyl43A
CK	100.0 ± 2.5	100.0 ± 1.6	100.0 ± 1.6
$NiCl_2$	126.5 ± 2.4	92.4 ± 0.6	4.2 ± 0.2
$CoCl_2$	115.9 ± 3.9	91.8 ± 1.0	0.0
NaCl	86.6 ± 2.4	97.6 ± 2.3	93.0 ± 2.5
$CrCl_3$	81.2 ± 3.1	107.3 ± 2.0	92.6 ± 2.7
$MgCl_2$	81.1 ± 3.0	94.4 ± 1.8	90.7 ± 1.6
$MnCl_2$	77.6 ± 3.4	87.4 ± 2.1	44.7 ± 0.8
KCl	76.0 ± 2.5	106.1 ± 3.1	92.9 ± 2.9
$CuCl_2$	76.0 ± 2.4	81.4 ± 2.4	0.0
$CaCl_2$	75.2 ± .9	100.7 ± 3.0	107.9 ± 3.0
$Pb(CH_3OO)_2$	66.2 ± 1.2	92.2 ± 2.9	54.0 ± 1.3
$ZnCl_2$	65.6 ± 2.8	96.1 ± 2.5	17.0 ± 0.4
AgNO	18.2 ± 0.9	70.0 ± 2.2	11.3 ± 0.9
β-Mercarptoethanol	102.3 ± 2.8	119.6 ± 1.2	102.0 ± 3.0
EDTA	76.1 ± 1.2	77.9 ± 2.7	7.0 ± 0.5
SDS	51.7 ± 2.3	0.0	4.4 ± 0.6

[a] Values represent the mean ± SD (n = 3) relative to the control samples (CK) without any addition.

Table S5. Primers used for qPCR analysis and gene cloning.

Name	Sequence (5′→3′) [a]
xyl43A-qF	CAAACTGAGCGACGACATGC
xyl43A-qR	TCGTCCAGCCTTTGACAGGC
xyl43B-qF	GATCCCCGAGTACGACAAGAC
xyl43B-qR	CGAACGATCCGATGTCCTCG
xyl43C-qF	GGTGGTAATTGGCTTCCAGGC
xyl43C-qR	CTATAATTTCGATCCGTATAAC
xyl43D-qF	GGTGGTAATTGGCTTCCAGGC
xyl43D-qR	CGACCTTGTATGTGACCCAGC
xyl43E-qF	CGTCTGTCTTTGGTAGGGAGTC
xyl43E-qR	CTCGAGATGCTGCGGTGTG
xyl43F-qF	CTTGATCAAATCACCAGATGGAAGC
xyl43F-qR	CAGTGTGAGCGGTATCCTTTGC
xyl43G-qF	CAGAGTCCTGGAGCATTGAGC
xyl43G-qR	CGCCCACAATGTCGAAGACG
xyl43H-qF	GACCCACCACCTGGAGAGATG
xyl43H-qR	GGTCGTAAGTGTTGAGCTCGG
xyl43I-qF	GTCTGTCCCCTTCGTGTGGG
xyl43I-qR	GTACCACCTTCAGCGGCTAG
xyl43J-qF	GCAATGAGGCACAGGGTGG
xyl43J-qR	GTGAACTGGACTTTGCTGGAC

xyn10A-qF	GGCGGAAGAGCGCAAGGCCCAG
xyn10A-qR	GACAAGGTTGTGGCAGTGGAT
xyn10B-qF	TGGTATCACCTCCTGGGGTA
xyn10B-qR	GCGTTGAGGAGAGCAGTGT
xyn10C-qF	CATTTCGAAGCAGTCATGC
xyn10C-qR	AGGAACAACTTGGCACTTGG
xyn10D-qF	GCGTGACAACATCTTCCTCTCG
xyn10D-qR	CTTCGAGGCCAAGGGCGGTGAAC
xyn10E-qF	GTGCTTCGCATGGGACGTCG
xyn10E-qR	GCGGCGAGACCGGTAAGTTTG
xyn11A-qF	CACCAATGGAGCCGGCAGCCG
xyn11A-qR	CTGCCATCGGAGGTGACGCTG
xyn11B-qF	CCGAATCTTGTCGTCAACTACAC
xyn11B-qR	GGTTGGTCCTTCGGACAGAC
xyn11C-qF	CGCCAAGACCGTCACCTAC
xyn11C-qR	CGGTGCTCCTTACGGACGG
xyn11D-qF	GCCAACAGAGTCATCAACTACAG
xyn11D-qR	GTGCCCTGGATGGAAGGCT
xyn11E-qF	GCGAGAGATATGCTGGTGAATTAC
xyn11E-qR	CGTTGATGCGCTTCTTCTGC
xyn11F-qF	GACTGACGGCCAGAGCGATG
xyn11F-qR	GTCCAGCCGTAGATGGCCAAG

xyn11G-qF	CAGGGAATGCTCTTCAACTACAC
xyn11G-qR	GCCAGACTTCGTAGGTGCC
xyn11H-qF	GCGCCAGGACCATCAAGTAC
xyn11H-qR	GACCAGTACTGGGTGAAGGTG
Tubulin-qF	GCGAGATTGTTCACCTCCAGAC
Tubulin-qR	GAACGTACTTGTTGCCGGATGC
xyl43A-F	GGGCATATGATGACCGACCCTACACAGAAAGACC
xyl43A-R	GGGGAATTCCTAGTGGTGGTGGTGGTGGTGCTCCTGCGGCTGCGCC
xyn10A-F	GGGCATATGAAGTGGGAGGTCATTGAGCTAAG
xyn10A-R	GGGGAATTCCTAGTGGTGGTGGTGGTGCTTACCACCCTTGATGCCCTTG
xyn11A-F	GGGCATATGGCCCCCCTCGACATCCTCACTG
xyn11A-R	GGGGAATTCCTAGTGGTGGTGGTGGTGAGACGTCTGAACGTAGATGTCAGCG

[a] For expression primers, the restriction sites are underlined, and each reverse primer contains a His6-tag-coding sequence.

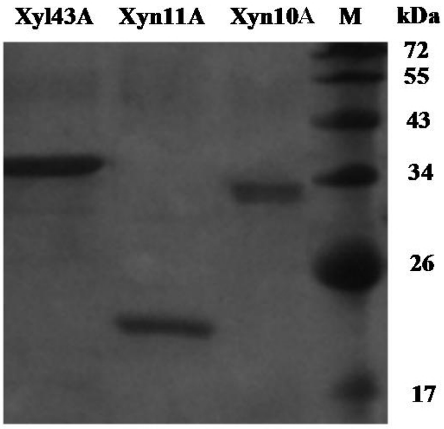

Figure S1. SDS-PAGE of the purified recombinant enzymes. M, the molecular weight markers.

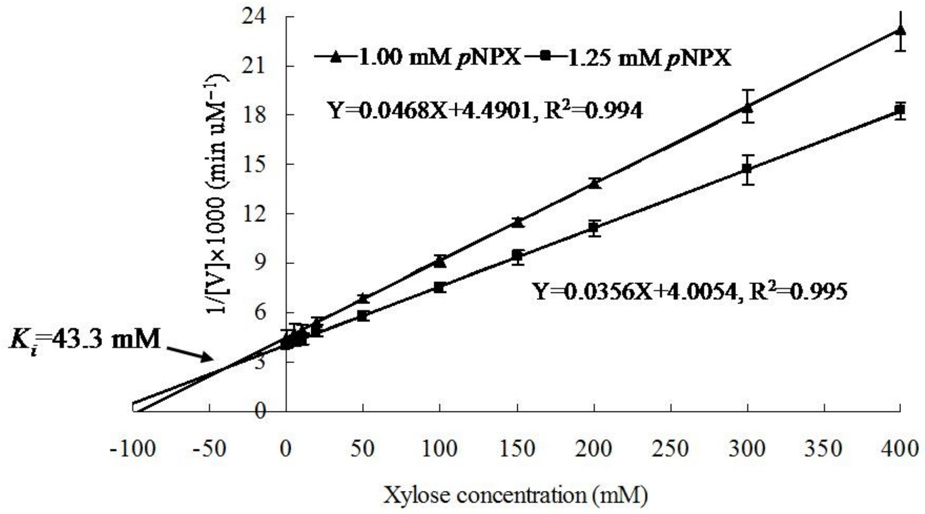

Figure S2. Xylose tolerance of the Xyl43A. *p*-Nitrophenyl-β-D-xylopyranoside (*p*NPX) at the concentrations of 1.00 mM and 1.25 mM were used as the substrates.

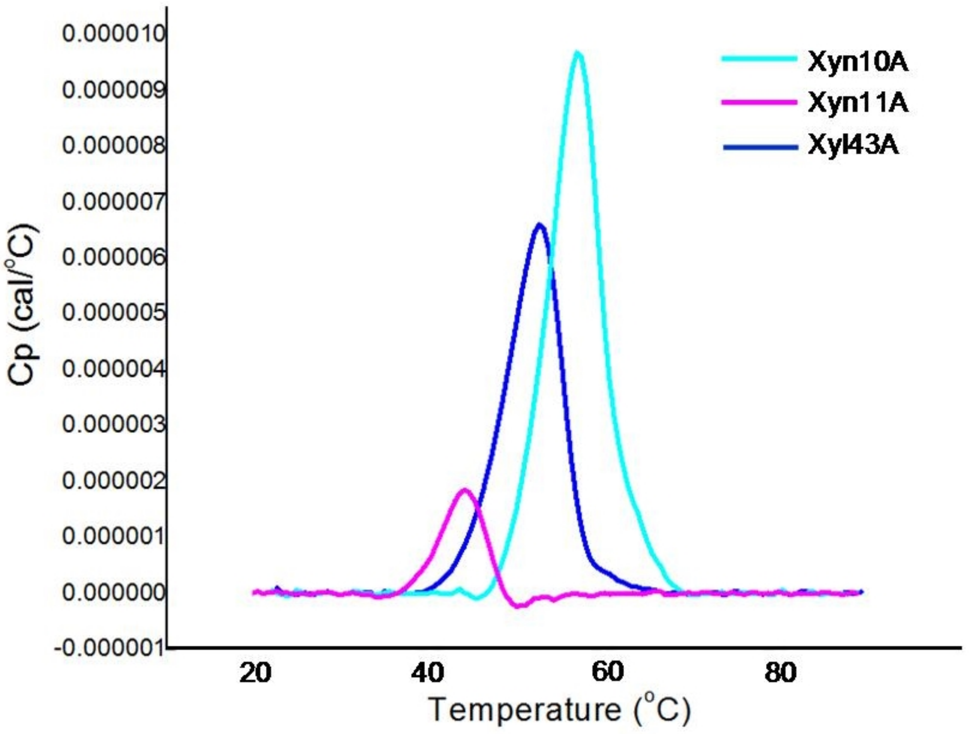

Figure S3. DSC analysis of the T_m values of the purified recombinant enzymes.
